# Lipoxin A4 Reduces Ventilator-Induced Lung Injury in Rats with Large-Volume Mechanical Ventilation

**DOI:** 10.1155/2020/6705985

**Published:** 2020-11-22

**Authors:** Qi Wang, Guang-xiao Xu, Qi-hang Tai, Yan Wang

**Affiliations:** Department of Anesthesiology, The Second Affiliated Hospital of Harbin Medical University, Harbin, China

## Abstract

Ventilator-induced lung injury (VILI) is a severe and inevitable complication in patients who require mechanical ventilation (MV) for respiratory support. Lipoxin A4 is an endogenous anti-inflammatory and antioxidant mediator. The present study determined the effects of lipoxin A4 on VILI. Twenty-four rats were randomized to the sham, VILI, and lipoxin A4 (LX4) groups. The rats in the VILI and LX4 groups received large-volume MV for 4 hours to simulate VILI. Capillary permeability was evaluated using the PaO_2_/FiO_2_ ratio, lung wet/dry weight ratio, and protein level in the lung. VILI-induced inflammation was assessed by measuring cytokines in serum and lung tissue, the expression and activity of NF-*κ*B, and phosphorylated myosin light chain. The oxidative stress response, lung tissue injury, and apoptosis in lung tissue were also estimated, and the expression of apoptotic proteins was examined. MV worsened all of the indices compared to the sham group. Compared to the VILI group, the LX4 group showed significantly improved alveolar-capillary permeability (increased PaO_2_/FiO_2_ and decreased wet/dry weight ratios and protein levels), ameliorated histological injury, and reduced local and systemic inflammation (downregulated proinflammatory factors and NF-*κ*B expression and activity). Lipoxin A4 notably inhibited the oxidative stress response and apoptosis and balanced apoptotic protein levels in lung tissue. Lipoxin A4 protects against VILI via anti-inflammatory, antioxidant, and antiapoptotic effects.

## 1. Introduction

Mechanical ventilation (MV) is an essential life support for patients in the ICU [1]. MV provides respiratory assistance and oxygen support, but it also causes severe complications, known as ventilator-induced lung injury (VILI) [2]. Approximately 24% of acute respiratory distress syndrome (ARDS) patients experience VILI in the ICU [3, 4]. The pathology of VILI is characterized by severe inflammation that is induced by repetitive cycles of stretching and the overinflation of alveoli, which directly injures endothelial and epithelial cells and disrupts alveolar-capillary permeability to result in lung oedema [5, 6]. Despite the use of the pharmacological treatment [7] and protective ventilation strategies [3], VILI still results in 40% mortality in ARDS patients [7].

Lipoxin and its receptor constitute an endogenous anti-inflammatory signal. Activation of the lipoxin receptor with an agonist may reduce VILI [8, 9]. Several subtypes of lipoxin exist, and lipoxin A4 protects against multiple organ injury via its anti-inflammatory, antioxidant, and antiapoptotic effects [10–13], and activated lipoxin A4 decreases the secretion of neutrophils and inhibits neutrophil adherence to the endothelium [14, 15]. According to these conclusions, we postulated that lipoxin A4 would attenuate VILI. However, no study directly investigated whether lipoxin A4 reduced VILI. The present study prepared a VILI model and administered lipoxin A4 to observe the effects of lipoxin A4 on VILI.

## 2. Materials and Methods

This study was approved by the Ethics Committee of Harbin Medical University. SD rats were purchased from the Animal Centre of Harbin Medical University. A total of 24 rats were randomly divided into sham, VILI, and lipoxin A4 (LX4) groups. Rats in the sham group received only anaesthesia and intubation. Rats in the VILI and LX4 groups received MV and an injection of saline or lipoxin A4. The MV parameters were based on a previous study [16] (tidal volume: 17 ml/kg, respiratory rate: 50/min, and inspiratory to expiratory ratio: 1 : 1), which were applied for 4 hours.

All rats were anaesthetized with 3% pentobarbital sodium (30 mg/kg) via intraperitoneal injection and intubated with a 14 G catheter. The femoral artery and vein were cannulated to analyse arterial blood gases and collect blood samples. MV was initiated after injection of 0.6 mg/kg rocuronium. Rats in the sham and VILI groups received 0.5 ml saline simultaneous with MV initiation, and the rats in the LX4 group received lipoxin A4 (100 *μ*g/kg diluted in 1 ml saline, administered via injection for 30 min) [10, 12] (Cayman Chemical, Ann Arbor, MI, USA). The anaesthesia was maintained with pentobarbital sodium (10 mg/kg intraperitoneally) and rocuronium (0.6 mg/kg) via intravenous injection at one-hour intervals. After 4 hours of ventilation, peripheral blood was collected from all rats, which were sacrificed using an overdose of anaesthetics. The heart and lungs were collected, and the right bronchus was clamped using a vessel clamp. Sterile saline (10 ml/kg) was injected into the left lung and withdrawn 5 times. Bronchoalveolar lavage fluid (BALF) was collected and centrifuged at 1000 g for 15 minutes at 4°C. The upper lobe of the right lung was collected and stored at -80°C. The middle lobe was collected and prepared for histological analysis. The lower lobe of the right lung was weighed and dried for 48 hours at 60°C, then reweighed to calculate the wet/dry weight ratio.

### 2.1. Alveolar-Capillary Permeability

To evaluate the effect of lipoxin A4 on alveolar-capillary permeability, we analysed arterial blood gases, calculated the wet/dry weight ratio, and tested the protein concentration in BALF. The PaO_2_ was measured at baseline and 4 hours after ventilation using a Bayer Rapidlab 348 (Bayer Diagnostics, Germany), and the PaO_2_/FiO_2_ was calculated. The lung tissue wet/dry weight ratio was calculated and recorded. Protein concentrations in BALF were investigated using the bicinchoninic acid (BCA) method.

### 2.2. Histological Examination

Lung tissue was prepared to histologically analyse injury using haematoxylin and eosin (HE) staining. Briefly, lung tissue was collected, fixed in 4% paraformaldehyde, and embedded in paraffin. The lung tissue was cut into 4 *μ*m sections and stained with HE. An independent pathologist evaluated and scored the histological injury according to pathological changes. The lung injury score was based on Table 1 and included lung haemorrhage, peribronchial infiltration of inflammatory cells, pulmonary interstitial oedema, pneumocyte hyperplasia, and intra-alveolar infiltration of inflammatory cells. The lung injury was scored between 0 and 10.

### 2.3. Inflammation

To assess the effects of lipoxin on VILI-induced inflammation, cytokines in serum and BALF were tested, and the numbers of macrophages and neutrophils in BALF were examined.

BALF was centrifuged, and the supernatant was collected to analyse the concentrations of TNF-*α*, IL-1*β*, IL-6, and IL-10 using ELISA kits (Wuhan Boster Bio-Engineering Limited Company, Wuhan, Hubei, China). The BALF sediment was collected to count the number of neutrophils and macrophages using Giemsa staining. The expression and activity of NF-*κ*B and the expression of phosphorylated myosin light chain (MLC) in lung tissue were also detected (Abcam, Shanghai, China).

Chemoattractants, including intercellular adhesion molecule- (ICAM-) 1 and macrophage inflammatory protein- (MIP-) 2, in serum were also measured using ELISA kits (Wuhan Boster Bio-Engineering Limited Company, Wuhan, Hubei, China). Serum (100 *μ*l) was added to each well for the testing of these indicators.

### 2.4. Oxidative Stress Response

A part of the lung tissue was collected to prepare homogenates with saline. The levels of cyclic guanosine monophosphate (cGMP) (581021-96S, Cayman Chemical, Michigan, USA) and MDA (10563-10, Cayman Chemical, Michigan, USA) and the activity of NADPH (9000743-50, Cayman Chemical, Michigan, USA) and superoxide dismutation (SOD) (Cayman Chemical, Michigan, USA) were determined using commercial kits. The expression of inducible nitric oxide synthase (iNOS) in lung tissue after ventilation was detected using Western blotting.

### 2.5. Apoptosis Assay

The level of apoptosis in lung tissue was tested using TUNEL staining and an apoptosis assay kit (Roche, Mannheim, Germany) according to the commercial instructions.

Briefly, lung tissue sections were digested with proteinase K then immersed in TUNEL reactive solution. Endogenous peroxidase activity was quenched with hydrogen peroxide, and the sections were immersed in an extra-avidin peroxidase and diaminobenzidine solution. After counterstaining with Mayer-haematoxylin and dehydration, brown nuclei were identified as positive for apoptosis. The sections were examined under a microscope, and the percentages of apoptotic lung cells were calculated. The expression of apoptotic proteins was investigated using Western blotting.

### 2.6. Western Blotting

Total protein was extracted from the lung tissue, and the protein levels were determined using a Bradford assay. The protein concentration was calculated, and 50 *μ*g of total protein was added to each well. The proteins were transferred to a polyvinylidene fluoride membrane. The membrane was blocked with fat-free milk and incubated with primary antibodies, including NF-*κ*B (sc-8414, Santa Cruz Biotechnology, CA, USA), p-MLC (3675S, CST), iNOS (ab178945, Abcam, Shanghai, China), Bax (ab32503, Abcam, Shanghai, China), Bcl-xL (ab32370, Abcam, Shanghai, China), and caspase-3 (ab13847, Abcam, Shanghai, China), and the dilution concentrations of these antibodies were 1 : 1000. After incubation with primary antibodies, the membrane was incubated with secondary antibodies. The bands were visualized using enhanced chemiluminescence.

### 2.7. Statistical Analysis

All of the data are expressed as the means ± standarddeviation(SD). The data were analysed using *t*-tests to compare differences between two groups. Continuous variables were analysed using one-way analysis of variance and post hoc Bonferroni correction. All of the data were analysed using SPSS Statistics 19.0 (SPSS, Chicago, IL, USA). A *P* value < 0.05 was considered statistically significant.

## 3. Results

### 3.1. Lipoxin A4 Improved Capillary Permeability in VILI

**Figure 1:**
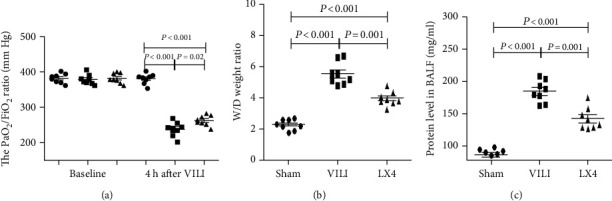
Lipoxin A4 improved alveolar-capillary permeability in VILI. Compared to the sham group, the PaO_2_/FiO_2_ was decreased, and the wet/dry weight ratio and protein levels in BALF were increased in the VILI and LX4 groups. Compared to the VILI group, the PaO_2_/FiO_2_ was increased (a), and the wet/dry weight ratio (b) and protein concentration (c) were decreased by lipoxin A4. ^∗^*P* < 0.05, vs. the sham group; ^#^*P* < 0.05, vs. the VILI group. ●: sham group; ■: VILI group; ▲: LX4 group.

The PaO_2_/FiO_2_, protein concentration, and wet/dry weight ratio were worse in rats that received ventilation than the sham group rats. Lipoxin 4 significantly increased the PaO_2_/FiO_2_ and decreased the protein concentration and wet/dry weight ratio in the LX4 group compared to the VILI group (Figure 1).

### 3.2. Lipoxin A4 Attenuated the Histological Injury Score in VILI

**Figure 2:**
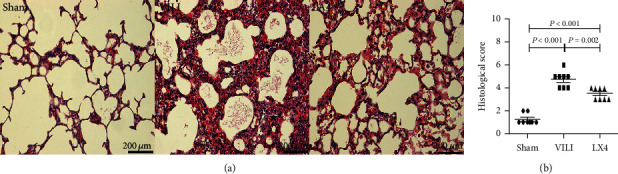
Lipoxin A4 reduced the histological injury score in VILI. After 4 hours of large-volume MV, many inflammatory cells infiltrated into lung tissue, and the alveolar wall was thickened and broken. There was severe mesenchymal oedema and haemorrhage in the lung tissue in the VILI group (a). Compared to the VILI group, the histological score in the LX4 group was significantly decreased. The histological injury score is presented in (b). ^∗^*P* < 0.05, vs. the sham group; ^#^*P* < 0.05, vs. the VILI group. ●: sham group; ■: VILI group; ▲: LX4 group.

Typical lung injury was observed in the rats that received MV for 4 hours. There were severe lung alveolar oedema and mesenchymal oedema in the VILI and LX4 groups. Many inflammatory cells infiltrated into the lung tissue, and there was haemorrhage in the mesenchyme. The alveoli in the VILLI group were significantly broken, and the alveolar wall was thickened. Compared to the VILI group, the LX4 group had significantly reduced histological injury (Figure 2).

### 3.3. Lipoxin A4 Reduced Inflammation after Mechanical Ventilation

**Figure 3:**
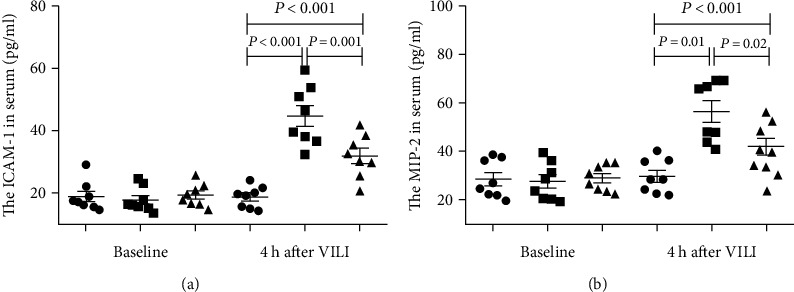
Lipoxin A4 reduced systemic inflammation in VILI. After MV, the levels of ICAM-1 and MIP-2 in serum were significantly increased in the VILI and LX4 groups. However, lipoxin A4 notably reduced the increase in ICAM-1 and MIP-2 levels in serum. ^∗^*P* < 0.05, vs. the sham group; ^#^*P* < 0.05, vs. the VILI group. ●: sham group; ■: VILI group; ▲: LX4 group.

We first determined the effect of lipoxin A4 on the serum levels of the chemoattractants ICAM-1 and MIP-2. ICAM-1 and MIP-2 levels in serum were significantly increased in the rats that received MV for 4 hours compared to the sham group. Compared to the levels in the VILI group, lipoxin A4 significantly reduced the ICAM-1 and MIP-2 (Figure 3).

**Figure 4:**
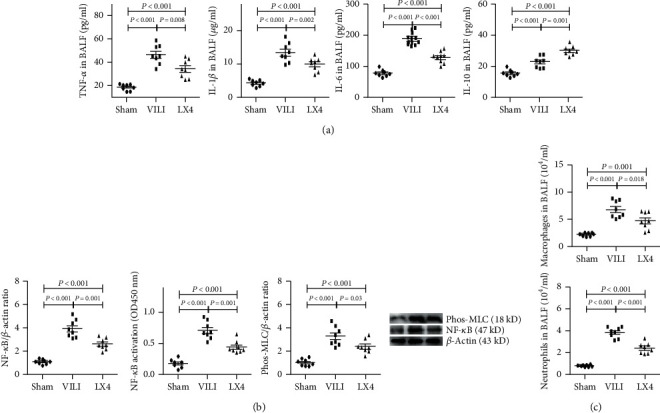
Lipoxin A4 inhibited VILI-induced local inflammation. Compared to the levels in the sham group, the levels of TNF-*α*, IL-1*β*, IL-6, and IL-10 were significantly increased in the VILI group after large-volume MV. Compared to the VILI group, TNF-*α*, IL-1*β*, and IL-6 levels were decreased, and the IL-10 level was increased in the LX4 group (a). The activity and expression of NF-*κ*B and the expression of p-MLC were notably increased in response to MV. The upregulation of NF-*κ*B and MLC was significantly abrogated by lipoxin A4 (b). Similar to the effect on cytokines, the increased infiltration of neutrophils and macrophages was also abrogated by lipoxin A4 (c). ^∗^*P* < 0.05, vs. the sham group; ^#^*P* < 0.05, vs. the VILI group. ●: sham group; ■: VILI group; ▲: LX4 group.

We also found that MV significantly upregulated TNF-*α*, IL-1*β*, IL-6, and IL-10 levels in the VILI and LX4 groups. Lipoxin A4 downregulated the expression of TNF-*α*, IL-1*β*, and IL-6 compared to the expression in the VILI group and upregulated IL-10 expression (Figure 4(a)). Lipoxin A4 also significantly inhibited the expression of NF-*κ*B and p-MLC and the activity of NF-*κ*B (Figure 4(b)).

Compared to the sham group, the numbers of neutrophils and macrophages in BALF were significantly increased after 4 hours of MV. Lipoxin A4 significantly decreased the number of neutrophils and macrophages compared to the VILI group (Figure 4(c)).

### 3.4. Lipoxin A4 Inhibited the Oxidative Stress Response

**Figure 5:**
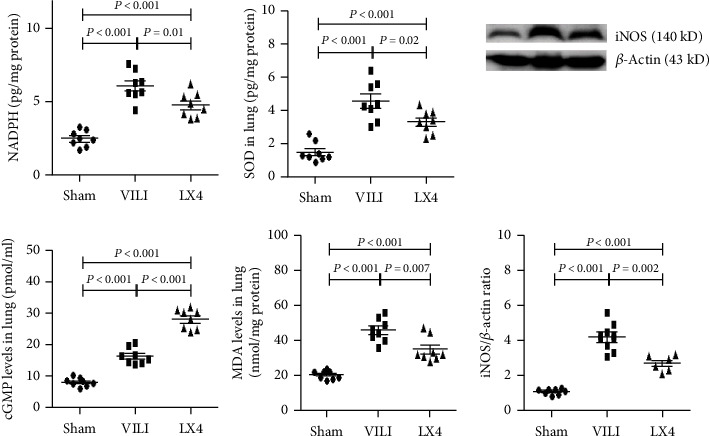
Lipoxin A4 reduced the oxidative stress response after VILI. In the VILI group, the activity of NADPH and SOD and the concentrations of cGMP and MDA were promoted by large-volume MV. Compared to the VILI group, NADPH and MDA were reduced by lipoxin A4, and cGMP and SOD were increased by lipoxin A4. ^∗^*P* < 0.05, vs. the sham group; ^#^*P* < 0.05, vs. the VILI group. ●: sham group; ■: VILI group; ▲: LX4 group.

After 4 hours of MV, cGMP and MDA levels and the activity of NADPH and SOD were significantly increased. Compared to the VILI group, the levels of cGMP and SOD were notably increased, and NADPH and MDA activity was decreased by lipoxin A4 (Figure 5). Lipoxin A4 also significantly decreased the expression of iNOS in lung tissue.

### 3.5. Lipoxin A4 Reduced Apoptosis after Ventilation

**Figure 6:**
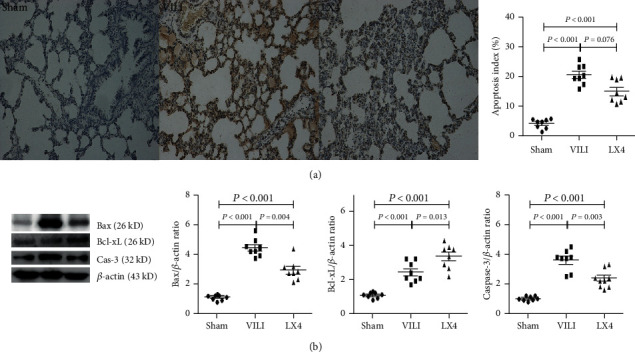
Lipoxin downregulated the apoptotic index in VILI. The number of apoptotic cells in lung tissue was increased in rats that received 4 hours of MV. Cellular apoptosis was significantly lower in the LX4 group than the VILI group (a). Compared to the VILI group, the LX4 group had a notably inhibited expression of Bax and caspase-3 but increased Bcl-xL expression in lung tissue (b). ^∗^*P* < 0.05, vs. the sham group; ^#^*P* < 0.05, vs. the VILI group. ●: sham group; ■: VILI group; ▲: LX4 group.

Many apoptotic cells were observed in the lung tissue of rats that received MV. Compared to apoptosis in the VILI group, apoptosis was notably reduced by lipoxin A4 in the LX4 group (Figure 6(a)).

The expression of apoptosis-regulated proteins was also detected. Lipoxin A4 decreased Bax and caspase-3 expression and increased Bcl-xL expression after MV (Figure 6(b)).

## 4. Discussion

The present study found that lipoxin A4 significantly reduced VILI. Lipoxin A4 notably improved capillary permeability, reduced local and systemic inflammation and the oxidative stress response, and decreased the apoptosis induced by large-volume ventilation.

MV is an important life support for patients with poor pulmonary function or critical illness. However, large-volume MV-induced physical forces may cause severe lung injury, which is characterized by pulmonary inflammation, infiltration of leucocytes, increased lung permeability, pulmonary oedema, and deterioration of pulmonary gas exchange [17]. Despite the application of lung protective strategies and other pharmacological treatments, VILI remains the major cause of death of individuals with ARDS [7]. Lipoxin A4 is an endogenous anti-inflammatory lipid mediator that is synthesised from arachidonic acid, and it attenuates different organ injuries via its anti-inflammatory, antioxidative, and antiapoptotic effects [12, 18, 19]. However, no study investigated the effect of lipoxin A4 on VILI. The present study injected lipoxin A4 into rats that received large-volume MV to evaluate its effects on VILI.

We first evaluated the effects of lipoxin A4 on VILI using functional and macroscopic indicators. After 4 hours of MV, lipoxin A4 significantly ameliorated histological injury. Lipoxin A4 also increased PaO_2_/FiO_2_, decreased the lung wet/dry weight ratio and total protein levels, and improved capillary permeability. These indicators suggested that lipoxin A4 notably attenuated VILI.

Local and systemic inflammation plays important roles during VILI. Under mechanical stretch activation, NF-*κ*B in the epithelium and endothelium is activated and promotes the release of proinflammatory factors [20] into the peripheral blood, which further induces inflammatory cell infiltration into the lung tissue [21]. Inflammatory cells further produce many cytokines, which results in lung injury [22]. The present study found that lipoxin A4 significantly reduced the infiltration of neutrophils and macrophages in the lung tissue, and this effect may result from lipoxin A4-mediated inhibition of the chemoattractants ICAM-1 and MIP-2 in the serum, which is similar to a previous study [23]. Lipoxin A4 decreased the infiltration of inflammatory cells [23–25] and downregulated release of the proinflammatory factors TNF-*α*, IL-1*β*, and IL-6 and inhibited VILI-induced local inflammation. According to the mechanism of inflammation, we hypothesised that the anti-inflammatory effect of lipoxin A4 primarily depended on NF-*κ*B. During inflammation, NF-*κ*B plays a central role in cytokine regulation, and blockade of NF-*κ*B significantly inhibited VILI [26]. The present study found that the expression and activity of NF-*κ*B were significantly increased, and lipoxin A4 significantly reduced NF-*κ*B expression and activity. We concluded that lipoxin A4 reduced inflammation primarily via the inhibition of NF-*κ*B [27]. MLC is activated in VILI pathology [28] and further disrupts the pulmonary endothelial barrier, which leads to lung oedema [29]. The inhibition of MLC may protect against VILI [30] and lung injury induced by endotoxin [29]. The present study found that lipoxin A4 significantly reduced p-MLC in lung tissue. This result indicated that lipoxin A4 may protect against VILI via the inhibition of MLC phosphorylation. In addition to inflammation, the oxidative stress response plays an important role in VILI. Reactive oxygen species are a major damage factor in VILI and trigger DNA damage [31]. During VILI, reactive oxygen species are produced by the endothelium, which is activated by mechanical stretching [32] and shearing stresses [33]. This production may be associated with the activation of iNOS in the endothelium and epithelium [34]. Activated neutrophils are another major source of reactive oxygen species. NADPH in activated neutrophils is significantly activated and produces reactive oxygen species [33]. The present study found that lipoxin A4 significantly reduced the expression of iNOS, the activity of NADPH, and MDA levels in lung tissue. In contrast to the effects of the oxidant, lipoxin A4 notably increased the activity of SOD and cGMP, which are important antioxidative species [33–35]. These results indicated that lipoxin A4 protected against VILI due to its antioxidative effect [36].

During the pathology of VILI, proinflammatory factors and reactive oxygen species result in apoptosis of the lung endothelium and epithelium. The severity of apoptosis primarily influences pulmonary function [37, 38]. During VILI, Bax and Bcl-xL play pivotal roles in apoptosis. Bax is a proapoptotic protein that promotes the activation of caspase-3, which cleaves DNA. In contrast, Bcl-xL blocks the release of Bax [39] and inhibits apoptosis [40]. The present study found that lipoxin A4 significantly inhibited MV-induced apoptosis. The antiapoptotic effect of lipoxin A4 was associated with the regulation of apoptotic proteins. Lipoxin A4 significantly reduced the expression of Bax and caspase-3 but increased the expression of Bcl-xL.

### 4.1. Limitation

The conclusions of this study are similar to previous studies and indicated that lipoxin receptor activation with an agonist mitigated VILI via the regulation of inflammation and apoptosis [8, 9]. These two studies lowered the novelty of the present research. However, the effect of receptor activation with the agonist on VILI is complex and mixed because several subtypes of lipoxin exist. Among these subtypes, lipoxin A4 is the most extensively studied. Compared to the two previous studies, we directly specified the therapeutic effect of lipoxin A4 on VILI via anti-inflammation and antiapoptosis. The second limitation of this study was that we did not explore the possible mechanism of lipoxin A4 on VILI. Our future studies will investigate the mechanism of lipoxin A4 on inflammation and apoptosis using a cellular model by applying a specific blocker or shRNA.

## 5. Conclusions

These results suggest that lipoxin A4 reduces large-volume MV-induced VILI. This protection was associated with the anti-inflammatory, antioxidant, and antiapoptotic effects of lipoxin A4.

## Figures and Tables

**Table 1:** Lung injury evaluation variables.

Parameter	Score
Haemorrhage	0 or 1
Peribronchial infiltration	0 or 1
Interstitial oedema	0 to 2
Pneumocyte hyperplasia	0 to 3
Intra-alveolar infiltration	0 to 3

## Data Availability

The datasets used and/or analysed during the current study are available from the corresponding author upon reasonable request.
